# Psychometric properties of the Italian body shape questionnaire: an investigation of its reliability, factorial, concurrent, and criterion validity

**DOI:** 10.1007/s40519-022-01503-6

**Published:** 2022-11-09

**Authors:** Enrica Marzola, Matteo Martini, Paola Longo, Federica Toppino, Francesco Bevione, Nadia Delsedime, Giovanni Abbate-Daga, Antonio Preti

**Affiliations:** Eating Disorders Unit, Department of Neuroscience, University of Turin, Via Cherasco 15, 10126 Turin, Italy

**Keywords:** Eating disorders, Anorexia nervosa, Body image, Body shape questionnaire, BSQ, Reliability, Validity

## Abstract

**Purpose:**

This study was set up to investigate the reliability, factorial, concurrent, and criterion validity of the Italian version of the 34-item Body Shape Questionnaire (BSQ) and its shorter versions.

**Methods:**

The study included 231 patients diagnosed with an eating disorder and 58 putatively healthy people (comparison sample). The Italian BSQ-34 was administered to participants together with the Hamilton Depression Rating Scale and the Hamilton Anxiety Rating Scale. Information on body mass index, caloric intake at baseline, and the number of episodes of self-vomiting per week was also acquired.

**Results:**

Cronbach’s alpha of BSQ-34 was 0.971 (95% confidence interval [CI] 0.965–0.976) in patients and 0.960 (0.944–0.974) in controls. Test–retest stability in patients (*n* = 69), measured with intraclass correlation coefficient, was 0.987 (0.983–0.991). Confirmatory factor analysis of the single-factor model yielded acceptable fit for all versions of the BSQ. On all BSQ versions, patients scored higher than controls with a large effect size when calculated as Cliff’s delta. BMI and mean caloric intake at baseline had a stronger association with BSQ-34 than levels of anxiety and depression. The analysis with the receiver operating characteristics (ROC) curve showed that the BSQ-34 distinguished patients with an eating disorder from controls with good accuracy (Area Under the Curve = 86.5; 95% CI 82.2–90.7).

**Conclusion:**

The Italian version of the BSQ possesses good psychometric properties, in both the long and the shortened versions, and it can be applied to measure body dissatisfaction for both clinical and research purposes.

**Level of evidence:**

Level III, Evidence obtained from well-designed cohort or case–control analytic studies.

**Supplementary Information:**

The online version contains supplementary material available at 10.1007/s40519-022-01503-6.

## Introduction

Body image dissatisfaction can be conceived as the negative attitude toward one’s physical appearance deriving from a perceived discrepancy between the actual physical appearance and the desired ideal state of the body [1]. Body image dissatisfaction is a core feature of eating disorders and often leads to overconcern with body shape and weight. A disturbance in the experience of body weight or shape and an excessive influence of body weight or shape on self-evaluation are diagnostic symptoms in the definition of anorexia nervosa, according to the American Psychiatric Association’s fifth edition of the diagnostic and statistical manual of mental disorders (DSM-5) [2]. An undue influence of body shape or weight on self-evaluation is also a diagnostic symptom for bulimia nervosa [2]. Although not listed as a diagnostic symptom, concerns with body shape and weight are frequently reported in patients with binge eating disorder as well [3]. Overall, there is evidence that overconcern with body shape and weight represents a risk factor for the development of an eating disorder [4–6]. Moreover, body image dissatisfaction was found to mediate the relationship between childhood/adolescence experiences of maltreatment—including sexual harassment and abuse—and the subsequent development of eating disorders [7–9].

Body dissatisfaction is part of a more complex body image disturbance in eating disorders, which includes an array of symptoms such as altered body self-perception, body uneasiness, and body-checking behavior [10]. Most investigation on body image disturbance in eating disorders has focused on body size estimation and on body dissatisfaction, the latter including both body appraisals and affective judgment [11]. Body size estimation is usually measured with analog scales, image marking, and optical distortion method [12]. Typically, in both anorexia and bulimia nervosa, patients tend to overestimate the size of their body [13]. Body misperceptions are a different kind of experience than body dissatisfaction, although body dissatisfaction might influence the way the body is perceived and estimated. Indeed, on a measurement level, body dissatisfaction can be conceived as the negative appraisal of one’s physical appearance and accompanying discontent. Several tools are available to measure body dissatisfaction. Some measures of body dissatisfaction are incorporated in larger questionnaires, such as the Weight and Shape Concerns Subscales of the Eating Disorders Examination Questionnaire (EDE-Q) [14], and the Body Dissatisfaction subscale of the Eating Disorder Inventory-3 (EDI-3) [15]. Internal consistency of these scales, especially when translated into another language, was found poor to fair, as a reflection of the translation validity that, when tested, was often found poor [11]. Other dedicated scales focus on the positive appreciation of the body, somehow a reverse construct of body dissatisfaction, such as the Body Appreciation Scale [16] and the Body Esteem Scale for Adolescents and Adults [17]. The Body Shape Questionnaire (BSQ [18]) is the earliest and one of the most used tools aimed at measuring body dissatisfaction, for both clinical and research purposes [19–21]. Particularly, the BSQ is a self-report questionnaire specifically aimed at assessing body dissatisfaction prompted by the feelings of being fat, an oft-reported feeling in people with, or at risk of, eating disorders [22–25]. This tool is based on 34 items rated on a six‐point scale, with a time interval focused on the past four weeks, where higher scores indicate greater dissatisfaction with body shape. The validity and reliability of the original BSQ-34 have been proved [26], and its psychometric properties have been confirmed for several of its translations [20, 27, 28]. Furthermore, the BSQ-34 has been also demonstrated to be sensitive to symptom change over time in response to treatment [25, 29]. Criterion validity (i.e., the degree to which the scores of the instrument are an adequate reflection of a ‘‘gold standard’’) was less often investigated for the BSQ-34 [18]). Over time, several shorter versions of the BSQ-34 have been proposed (see, for details: [30, 31]). However, all versions retained a mono-factorial structure, suggesting that the tool measures just one broad construct of body dissatisfaction.

Today, several measures of body dissatisfaction or appreciation are available in the Italian language, such as the scales incorporated in the EDI-3 [32] or in the latest version of the EDE-Q [33]. A validated Italian version exists of the Body Appreciation Scale [34] and of the Body Esteem Scale for adolescents and adults [35]. Compared to other tools, the BSQ-34 explores a more extended set of feelings and behaviors related to body image, ranging from excessive concerns about weight and shape to embarrassment in public, the avoidance of certain activities and of exposure of one’s own body, the feelings of fatness experienced after eating. The conceptualization of body dissatisfaction as a multilayered construct might be helpful in different clinical sets involved in the diagnosis and treatment of eating disorders within the fields of psychology, psychotherapy, medicine, dietetics, and food sciences, for both the purposes of assessment of severity and the evaluation of the response to treatment. Moreover, the detailing of the construct, as implemented in the BSQ-34, allows its application to measure concerns about body weight and shape well beyond anorexia and bulimia nervosa, such as in people with obesity, independently from the comorbidity with binge eating disorder [26, 36]. However, despite its large use in Italy [37–39], the psychometric properties of the Italian version of the BSQ-34 have never undergone formal evaluation. So far, only the 14-item version of the BSQ has been investigated in Italy and proved to possess a mono-factorial structure, optimal internal consistency (with Cronbach’s alpha = 0.93), and a good convergent validity with independent measures of body dissatisfaction, in a sample of 497 non-clinical young women [40]. In the Matera et al. [40] paper, the lack of a formal validation study of the Italian version of the BSQ-34 was indeed indicated as a gap to fill.

### Aim

Since for both clinical use and research purposes, it is necessary to use tools with established reliability and validity [41], this study was set up to investigate the reliability, and the factorial, concurrent and criterion validity of the Italian version of the BSQ-34 and of its shorter versions.

## Methods

The study has been conducted according to the 1995 Declaration of Helsinki and its revisions [42]. This study was approved by the Ethical Committee of the local academic hospital. All participants signed a written informed consent.

### Participants

The study included both patients diagnosed with an eating disorder and putatively healthy people, who were enrolled as a comparison sample (hereinafter, controls).

All consecutive admissions among those seeking voluntary hospitalization at a major academic Eating Disorders Center were invited to take part in the study. Overall, 231 patients were invited to take part in the study. The following inclusion criteria were applied to the patients’ group: (a) diagnosis of an eating disorder as assessed by an experienced psychiatrist with the Structured Clinical Interview for Diagnostic and Statistical Manual of Mental Disorders, 5th edition (SCID-DSM-5) [43]; (b) age > 18 years old; and (c) no current or past psychotic or bipolar disorders or current drug abuse. All patients were offered an integrated and multidisciplinary intervention, including staff from different disciplines (psychiatry, clinical psychology, nursing, nutrition and dietetics, internal medicine). The normalization of the eating patterns and achieving higher motivation to comply with the next treatment steps were the main goals of inpatient treatment.

Controls were enrolled among people from the general population living in the same town, and they were recruited via advertisement and by word of mouth. The inclusion criteria for controls were: (a) the absence of a personal history of any psychiatric disorder or consultation in psychiatry; (b) age > 18 years old; and (c) the absence of a family history of an eating disorder in a first-degree relative.

Overall, 58 controls took part to the study. The sample size of controls was based on the calculation of the minimum sample size necessary for a receiver operating characteristics (ROC) curve analysis. With alpha set at 0.05 and power at 80% (beta = 0.20), assuming a 1-to-4 ratio in controls to cases, 15 controls against 58 cases were enough to perform a ROC analysis able to detect an AUC of at least 0.70 (the minimum fair area under the curve [AUC], see below). The calculation was based on the easyROC web-tool (http://www.biosoft.hacettepe.edu.tr/easyROC/) [44]. Based on the mean admission rate per month, we were expecting to enroll about 235 cases over the time interval of the study. Thus, we aimed to recruit 60 controls, and eventually we recruited 58 controls, which is the perfect match for a 4-to-1 ratio for our 231-patient sample size.

### Measures

All participants were administered a sociodemographic schedule providing information on gender, age and education, and they were asked to fill in the Italian version of the BSQ-34. Specifically, the BSQ-34 was translated into Italian by a psychiatrist fluent in English with 10 years of experience in eating disorders, and it was independently cross-checked by a senior psychiatrist fluent in English with over 25 years of experience in eating disorders; the Italian version of the BSQ-34 was further retro-translated into English by a native speaker and compared to the original version for meaning and form. The final version of the Italian BSQ-34 was then finalized with the aid of a third experienced psychiatrist fluent in English.

Patients were also inquired about levels of anxiety and depression, height and weight, mean caloric intake at baseline, and number of episodes of self-vomiting per week at baseline. Specifically, levels of depression were measured with the 17-item Hamilton Depression Rating Scale (HDRS) [45], and levels of anxiety were measured with the 14-item Hamilton Anxiety Rating Scale (HARS) [46].

Particularly, the HDRS aims at measuring the presence and the severity of symptoms of depression and it includes 17 items, nine of which rate from 0 (absent) to 4 (severe), and eight of which rate from 0 (absent) to 2 (clearly present). The scoring of the HDRS derives from a semi-structured interview carried out during the diagnostic assessment. The HARS aims at measuring the presence and the severity of symptoms of anxiety and it includes 14 items, each rated on a scale from 0 (absent) to 4 (severe). As for the HDRS, the scoring of the HARS was based on a semi-structured interview during the diagnostic assessment.

The body mass index (BMI) was calculated as the weight in kilograms divided by the square of the height, in meters. The caloric intake at baseline was expressed as the number of kilocalories taken per day (kcal/day) by the individual on a typical day in the month prior to the assessment. Particularly, this information was declared by the individual at the SCID evaluation, and it was confirmed by the clinician based on the standard conversion into calories of the reported eaten food. The number of episodes of self-vomiting per week was calculated as the mean of weekly episodes of self-induced vomiting in the last four weeks as declared by the individual at the SCID evaluation.

### Statistics

Data were entered in Excel, then coded and analyzed using the Statistical Package for Social Sciences (SPSS) version 27. Specific analyses were done with dedicated packages running in R [47]. All tests were two tailed, with alpha set at *p* < 0.05.

Descriptive statistics were reported as means with standard deviation and median with interquartile range, when appropriate, or as counts and percentages. Non-parametric tests were used to assess differences between groups or correlations among variables, except for age. Cliff’s delta was used to estimate effect size in the differences between cases and controls since it is considered more apt in non-parametric analysis [48]. On the basis of a rule-of-thumb, Cliff’s delta values are suggested to be small at ≥ 0.11; medium at ≥ 0.28; large at ≥ 0.43 [49].

Floor and ceiling effects were calculated to assess the scale attenuation effects [50]. The occurrence of floor or ceiling effects indicates that extreme items are missing in the lower or upper end of the scale, hence indicating a limited content validity. When 15% or more of respondents scored at the minimum (in this case, zero) or the maximum scores, the floor or ceiling effect was considered to have occurred.

Reliability was measured as internal coherence using Cronbach’s alpha, with 95% confidence interval as derived from a two-way mixed design analysis of variance. According to a shared rule-of-thumb, a threshold > 0.7 was set to consider the reliability as “good”, and values > 0.9 can be considered “excellent” [51]. Test–retest stability of the BSQ was evaluated in a subgroup of 69 patients (out of 231), who were invited to complete the BSQ again after a period around 3–6 weeks (median: 4 weeks). The intraclass correlation coefficient (ICC), with a 95% Confidence Interval (CI), was used to measure test–retest stability. According to a rule-of-thumb, ICC values ≥ 0.60 are considered acceptable for clinical use [52]. We also applied a graphical test to evaluate the reproducibility of repeated measurements in the same population [53]. According to Bland–Altman’s method, we plotted the difference between test- and retest scores against the mean of test- and retest scores for each participant. Confidence intervals for the mean difference were also calculated to determine whether the mean difference deviates significantly from zero, which should not. We adapted a pre-existing ad hoc code running in R to draw the Bland–Altman plot [54].

Confirmatory Factor Analysis (CFA) was applied to the items of the BSQ to make sure that a single global score was an appropriate summary measure of the screeners in the total sample. The Diagonally Weighted Least Squares (DWLS) estimator and a mean- and variance-adjusted chi-squared test statistic, aka weighted least square mean and variance-adjusted (WLSMV) estimator, were used in CFA since scores were ordinal, and the Mardia’s test [55] revealed a violation of multivariate normality in the data (skew = 12,203; *p* < 0.0001; small sample skew = 12,371; *p* < 0.0001). Indeed, there is evidence that the DWLS is superior to robust maximum likelihood even when the normality assumption is slightly or moderately violated [56]. The following parameters were used for fit estimation: the chi-square, the Comparative Fit Index (CFI), the Root Mean Square Error of Approximation (RMSEA), and the Standardized Root Mean Square Residual (SRMR). Even in the presence of chi-square with *p* < 0.001, according to the conventional rules of thumb [57], RMSEA values of 0.08 or lower, SRMR values of 0.09 or lower, and CFI values of 0.90 or higher were considered as an indication of acceptable fit. Items were retained in the model when their loading on the unidimensional factor was ≥ 0.40 [58]. Both the original (i.e., 34-item version [18]) and the several short forms of the BSQ (i.e., 16-item, 14-item, and 8-item versions; [30]) were tested (details on factor structure in Pook et al. [31]). As in past studies, a single-factor solution was tested, since this is the structure that dominates past analysis and the questionnaire is scored as such [19, 26]. A sample size of 200 participants is enough to test with CFA simple models as those tested in this study [59].

Cronbach’s alpha and McDonald’s omega, as estimated from the model, were also reported. McDonald's omega is considered a realistic estimate of the true reliability of a scale [60].

Once the best version of the BSQ was established via CFA, we tested the concurrent and criterion validity of the tool. For concurrent validity, we tested whether indicators of eating disorder pathology and of general psychopathology were associated with the scores on the BSQ. We expected the indicators of eating disorder pathology to have a stronger association with the BSQ scores than the indicators of general pathology. Spearman’s *ρ* was used to test the association between variables, and Fisher’s Z-transformation via Steiger’s test was used to compare dependent correlations. There is evidence that applying the Fisher transformation to the Spearman correlation coefficients for testing the equality of coefficients is justified when data are nonnormal [61]. The following variables were used to test the concurrent validity: the BMI, the mean caloric intake at baseline, and the number of episodes of self-induced vomiting per week at baseline, as indicators of eating disorder pathology; the total scores on the HARS and on the HDRS as indicators of general psychopathology.

We also assessed the criterion validity of the BSQ—i.e., the degree to which the scores of the instrument were an adequate reflection of a ‘‘gold standard’’ [62]. Specifically, for the purposes of this study we used the diagnosis assigned after the SCID interview as a “gold standard” for reference (having an eating disorder versus not having a mental disorder). The criterion validity of the BSQ was tested with the Receiver Operating Characteristics (ROC) curve. The following parameters were calculated: sensitivity, specificity, positive predictive value (PPV), negative predictive value (NPV), and positive diagnostic likelihood ratio. Sensitivity was defined as the probability of a true positive case—i.e., the probability of identifying a patient with an eating disorder. Specificity was the probability of a true negative case—i.e., the probability of identifying a patient without an eating disorder. The PPV was defined as the probability that a person is an eating disorder case when a positive test result is observed; the NPV was defined as the probability that a person is not a case of an eating disorder when a negative test result is observed; and the positive diagnostic likelihood ratio was calculated as the odds ratio that a positive test is observed in a population of people with an eating disorder compared to the odds that the same result is observed among a population of people without an eating disorder. The area under the curve (AUC; with 95% confidence interval) was used to measure the accuracy of the prediction. According to a shared rule-of-thumb, values of AUC ≤ 0.70 indicate poor accuracy; between 0.70 and 0.80, fair; between 0.80 and 0.90, good; above 0.90, excellent [63]. The “pROC” package running in R was used to perform the ROC analysis [64]. The best cut-off point for the BSQ was established according to the Youden’s method [65] with the “OptimalCutpoints” package running in R [66].

## Results

The sample included 231 patients diagnosed with an eating disorder and 58 controls. Table 1 details the most relevant characteristics of the sample. The sample included predominantly female participants, as a reflection of the greater prevalence of eating disorders among women. Mean age did not differ between patients and controls, but the age range was larger among patients. Patients had a lower level of education than controls, measured as years of completed education, and as expected, a lighter BMI. The clinical sample included predominantly patients with anorexia nervosa (171 out of 231), mostly of the restrictive type. Duration of illness was on average 6 years, with a large range from 0 to 42. In the patients’ sample, the duration of the illness was shorter than 1 year in 13 patients (6%); it was 1–3 years in 94 patients (roughly a half, 44%); 4–9 years in 63 of them (29%); and 10 years or longer in 45 patients (21%).

**Table 1:** General characteristics of the sample

	Patients	Controls	Statistics
Gender			
Males	13 (6%)	5 (9%)	*χ*^2^ = 0.18; d*f* = 1; *p* = 0.67
Females	205 (94%)	53 (91%)	
Age (years)			
Mean (SD)	25 (4)	24 (8)	Welch’s *t* = 0.89; d*f* = 89.9; *p* = 0.37 Mann–Whitney *U* test: *z* = − 2.78; *p* = 0.005
Range	16–56	18–35	
Education (years completed)			
Mean (SD)	13 (3)	16 (2)	*t* = 3.92; d*f* = 240, *p* < 0.0001
Range	8–25	12–18	
Body mass index (kg/m^2^)			
Mean (SD)	15.7 (4.6)	20.6	*t* = 6.08; d*f* = 251; *p* < 0.0001
Range	9.8–48.8	(2.2) 17.3–27.3	
Caloric intake at baseline			
Mean (SD)	790.3 (576.7)		
Range	0–5000		
Number of episodes of self-induced vomiting per week			
Mean (SD)	4 (14)		
Range	0–14		
Hamilton depression rating scale			
Mean (SD)	18.7 (7.5)		
Range	1–53		
Hamilton anxiety rating scale			
Mean (SD)	18.8 (7.5)		
Range	2–42		
Diagnosis			
AN-R	128 (55.4%)		
AN-BP	43 (18.6%)		
BN	20 (8.7%)		
Others EDs	40 (17.3%)		

### Reliability of the BSQ-34 in the sample

Cronbach’s alpha of BSQ-34 was 0.971 (95% CI 0.965–0.976) in patients and 0.960 (0.944–0.974) in controls. Test–retest stability in patients (*n* = 69), measured with ICC, was 0.987 (0.983–0.991). Test–retest stability was not measured in controls.

**Fig. 1:**
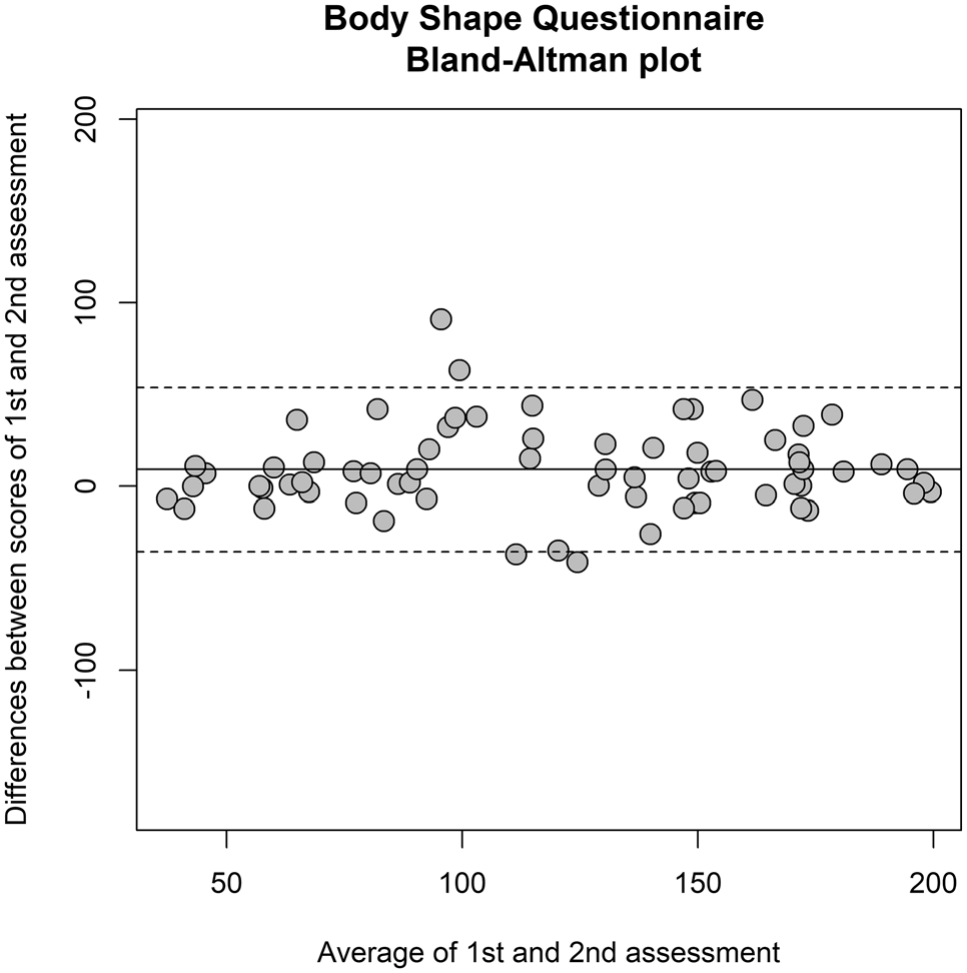
Bland–Altman plots for the Italian BSQ-34. The y-axis represents the change of BSQ-34 scores from the first measurement to the second (test and retest) and the x-axis is the mean of BSQ-34 scores of first and second measurements. Center line is the mean change of the score, while the upper and lower lines represent the limits of agreement for 95% confidence intervals

After 3–6 weeks of treatment (median: 4 weeks), the mean difference between the first and the second assessment of the BSQ in the 69 participants of the patients’ group was 9.1 (SD = 22.7). The 95% CI for the mean difference was − 14.6 to − 3.6 (since 0 is not within the confidence interval, the mean difference statistically differs from 0, suggesting that a change in the BSQ-34 is observed after treatment). However, by plotting the differences and the means of the two assessments in the Bland–Altman plot, only 3 cases out of 69 (4.3%) were outside the upper and the lower limits of agreement (Fig. 1).

### Confirmatory factor analysis (CFA) of the BSQ

CFA was tested in the patients’ group (*n* = 231). The fit was rejected for all models based on the chi-squared test. It was acceptable for the original BSQ-34, on the basis of CFI, RMSEA, and SRMR. The fit was suboptimal, based on the RMSEA, but still acceptable, based on CFI and SRMR, for the shorter versions of the BSQ. There was a further modest decay of reliability in the 8-item different versions (Table 2).

**Table 2:** Confirmatory factor analysis of the BSQ. Goodness-of-fit indices of the tested models with mean- and variance-adjusted chi-squared test statistic

Model	*χ*^2^	d*f*	*p*	CFI	RMSEA (90% CI)	SRMR	Cronbach’s *α*	McDonald’s *ω*
BSQ-34	959.1	527	0.0001	0.917	0.060 (0.054–0.066)	0.060	0.971	0.972
BSQ-16 A	236.8	104	0.0001	0.940	0.075 (0.062–0.087)	0.052	0.944	0.947
BSQ-16 B	265.0	104	0.0001	0.930	0.082 (0.070–0.094)	0.057	0.944	0.948
BSQ-14	225.5	77	0.0001	0.924	0.092 (0.078–0.106)	0.058	0.939	0.943
BSQ-8 A	68.8	20	0.0001	0.943	0.103 (0.077–0.130)	0.052	0.887	0.895
BSQ-8 B	49.1	20	0.0001	0.967	0.080 (0.052–0.108)	0.045	0.894	0.897
BSQ-8 C	64.5	20	0.0001	0.942	0.098 (0.072–0.126)	0.052	0.878	0.892
BSQ-8 D	53.5	20	0.0001	0.970	0.085 (0.058–0.113)	0.044	0.904	0.910
Threshold for fit			> 0.05	> 0.90	< 0.08	< 0.09	≥ 0.70	≥ 0.90

The original 34-item version had a good fit to the data, with just one item (item 33) showing factor loading lower than the predefined threshold of 0.400 (Table A1 in Supplementary material). All subsequent analyses were done with the BSQ-34, since there were no reasons to prefer other versions of the tool.

### Distribution of the scores of BSQ in the sample

There were no floor and ceiling effects for the BSQ-34. In the sample, 2 patients (1%) and 3 controls (5%) scored 34 (the minimum score) on the BSQ. No participants scored 204 (the maximum score) on the BSQ.

Overall, patients scored higher than controls on the BSQ-34, with a large effect size when calculated as Cliff’s delta (Table 3). All other shorter versions of the BSQ retained the ability to differentiate patients with eating disorders from controls at a large effect size (Table 3).

**Table 3:** Distribution of scores of the BSQ in patients and controls according to its versions

	Patients (*n* = 231)	Controls (*n* = 58)	Mann–Whitney *U* test	Cliff’s delta (95% CI)
BSQ-34	123.9 (45.7)	62.1 (22.2)	*z* = − 8.59; *p* < 0.0001	0.729 (0.634–0.803)
BSQ-16 A	58.9 (22.2)	29.9 (10.7)	*z* = − 8.35, *p* < 0.0001	0.709 (0.611–0.786)
BSQ-16 B	60.3 (22.2)	29.9 (11.5)	*z* = − 8.62, *p* < 0.0001	0.732 (0.635–0.806)
BSQ-14	54.1 (19.9)	27.7 (11.2)	*z* = − 8.37, *p* < 0.0001	0.711 (0.609–0.790)
BSQ-8 A	28.7 (11.0)	15.2 (5.7)	*z* = − 7.98, *p* < 0.0001	0.678 (0.574–0.760)
BSQ-8 B	30.2 (11.6)	14.7 (5.3)	*z* = − 8.57, *p* < 0.0001	0.728 (0.632–0.802)
BSQ-8 C	31.7 (10.7)	15.3 (6.1)	*z* = − 9.12, p < 0.0001	0.774 (0.684–0.840)
BSQ-8 D	28.6 (11.9)	14.7 (5.7)	*z* = − 7.72, p < 0.0001	0.655 (0.548–0.740)

### Concurrent validity

The BMI and the number of episodes of self-vomiting per week were positively, and the mean caloric intake at baseline was negatively associated with the scores on the BSQ-34 (Table 4, top). Levels of anxiety and depression were modestly and positively associated with the scores on the BSQ-34. Overall, the association of the BSQ-34 with the BMI on one side, and with the mean caloric intake at baseline on the other side, were statistically stronger than the association with the other indicators (Table 4, bottom).

**Table 4:** Correlations between BSQ scores and indicators of eating disorder pathology and of general psychopathology and Steiger’s test of BSQ versus variables at intersection

Spearman’s *ρ*	BMI	Caloric intake	Episodes of self-induced vomiting	HARS	HDRS
BSQ	*ρ* = 0.483 *p* < 0.0001	*ρ* = − 0.213 *p* = 0.004	*ρ* = 0.316 *p* < 0.0001	*ρ* = 0.167 *p* = 0.024	*ρ* = 0.160 *p* = 0.030
BMI		*ρ* = − 0.136 *p* = 0.071	*ρ* = − 0.347 *p* < 0.0001	*ρ* = − 0.178 *p* = 0.016	*ρ* = − 0.170 *p* = 0.021
Caloric intake			*ρ* = − 0.124 *p* = 0.099	*ρ* = − 0.022 *p* = 0.781	*ρ* = − 0.057 *p* = 0.465
Self-induced vomiting				*ρ* = 0.096 *p* = 0.197	*ρ* = 0.119 *p* = 0.110
HARS					*ρ* = 0.612 *p* < 0.0001
Steiger’s test					
BMI		*z* = 8.15 *p* < 0.0001	*z* = 2.22 *p* = 0.024	*z* = 3.64 *p* = 0.0003	*z* = 3.70 *p* = 0.0002
Caloric intake			*z* = − 4.91 *p* < 0.0001	*z* = − 3.62 *p* = 0.0003	*z* = − 3.49 *p* = 0.0005
Episodes of self-induced vomiting				*z* = 1.56 *p* = 0.118	*z* = 1.65 *p* = 0.098
HARS					*z* = 0.11 *p* = 0.913

### ROC analysis

**Fig. 2:**
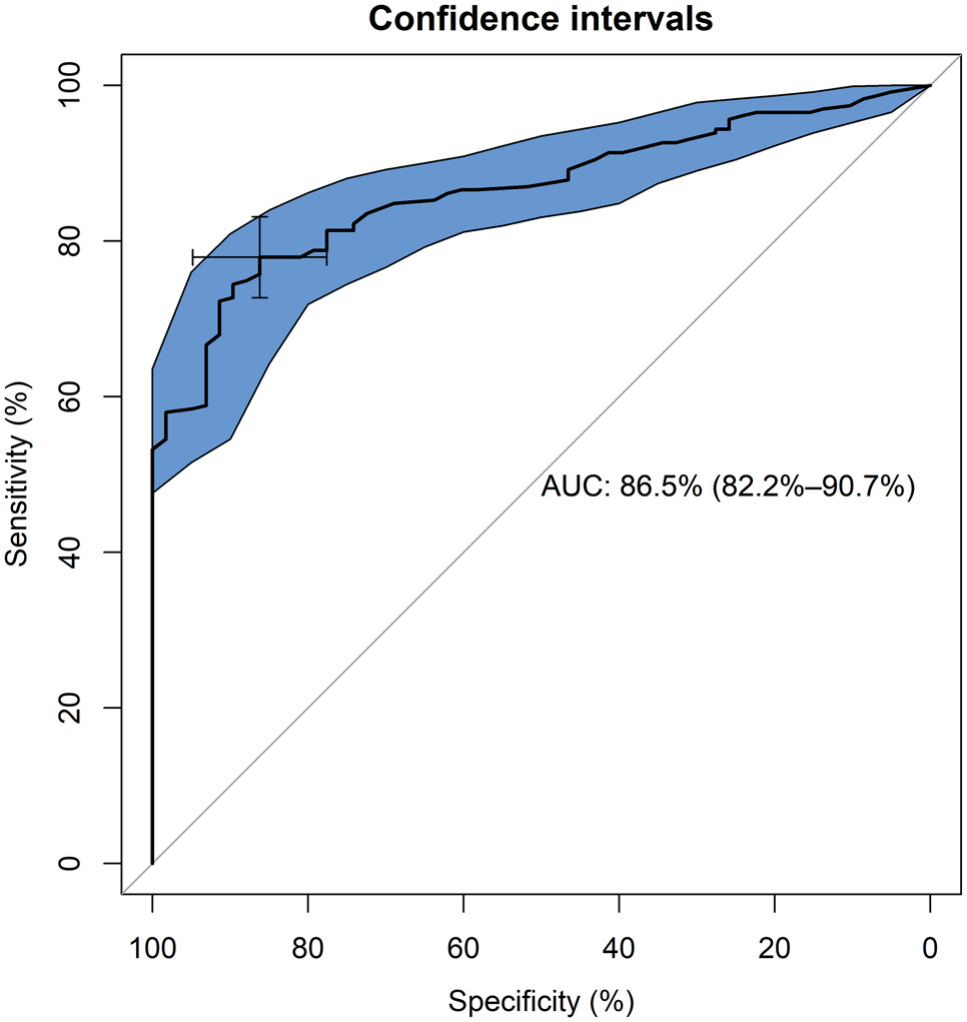
Receiver operator characteristic (ROC) curve of the predictive capacity of the Italian BSQ-34 in differentiating patients with eating disorders from healthy people (controls). Sensitivity and specificity are reported as percentages, with a cross on the curve indicating the best compromise between them. The area under the ROC curve (AUC) is reported alongside its 95% confidence interval

The BSQ-34 was able to distinguish patients diagnosed with an eating disorder from controls, with good AUC 86.5; 95% CI 82.2–90.7 (Fig. 2).

The best threshold for the differentiation of patients with eating disorder from controls was 81. Sensitivity and specificity at the best threshold were 78% and 86%, respectively. BSQ-34 had a better PPV (95.7%) than NPV (49.5%). The positive diagnostic likelihood ratio was 5.6.

In the investigated samples, 8 controls (14%) and 180 patients with an eating disorder (78%) scored at or above the cut-off on the BSQ-34 (*χ*^2^ = 81.06; d*f* = 1; *p* < 0.0001).

When the analysis was limited to the patients with diagnosis of AN or BN, those with BN or AN-BP were more likely to score above the best threshold on the BSQ-34 than those with AN-R: respectively, 19 out of 20 (95%), 39 out of 43 (91%), and 89 out of 128 (70%); *χ*^2^ = 12.23; d*f* = 2; *p* = 0.002.

## Discussion

The Italian version of the BSQ showed good to excellent psychometric properties in this study. The reliability in the sample was excellent or reasonably good for all versions of the BSQ, with the longer versions showing a better internal consistency than the shorter ones. The test–retest stability of the BSQ-34 was also excellent in the subsample of patients who were involved in the measurement. All involved patients were in treatment, and the scores of the BSQ-34 underwent a decrease, with evidence of sensitivity to change of the tool, as shown in past studies [25, 29]. However, only a minority of participants involved in the test–retest measurement underwent changes large enough to be positioned outside the main trend in the sample.

For all versions of the BSQ, a single-factor solution was tested with fair to excellent fit, suggesting that the tool measures a global construct of body dissatisfaction induced by the feeling or the fear of being fat. The specificity of the BSQ to measure this construct is helpful in the perspective of assessing changes in the core symptoms of eating disorders. However, it should be considered that body avoidance and checking are important psychological dimensions in eating disorders at both ends as well [25, 67–69], and they may contribute to the onset, course, and outcome of these diseases [70, 71]. Thus, the use of the BSQ for clinical purposes might be supplemented with likewise specific measures of body checking and avoidance or it might be complemented with multidimensional tools, such as the Body Uneasiness Test (BUT; [72]). The BUT is indeed aimed at measuring several experiences of body uneasiness, such as body shape and/or weight dissatisfaction, avoidance, compulsive control behaviors, detachment and estrangement feelings toward one’s own body, and specific worries about particular body parts, shapes, or functions [72, 73].

Nevertheless, the psychometric properties of the BSQ are excellent, as found in this and past studies, and it may be a valid tool for the assessment and outcome measurement. The lack of floor and ceiling effects supports the good content validity of the BSQ-34. Scores on the BSQ-34 were able to distinguish individuals with and without eating disorder and they correlated with measures of both general and eating disorder specific psychopathology. Indeed, higher scores on the BSQ-34 were associated with increasing levels of depression and anxiety, suggesting that the body dissatisfaction that is measured by the tool is partially correlated with psychological distress. Moreover, and as expected according to the cognitive-behavioral theory [4, 22], increased body dissatisfaction as measured by the BSQ-34 was related to (and possibly led to) conducts aimed at changing the body weight and shape, such as restrictive eating—as measured by the caloric intake—and self-induced vomiting. Finally, criterion validity of the BSQ-34 was proved by its ability to differentiate patients with eating disorders from controls with good accuracy when measured with AUC. Given its PPV and NPV, the BSQ-34 was more apt at identifying someone with an eating disorder than at excluding its presence. However, this might depend on the greater prevalence of cases with an eating disorder in the total sample, with only a minority being controls without an eating disorder.

Overall, these findings are congruent with the results of past studies on the original and the translated versions of the BSQ. Because of its high internal reliability and its robust single-factor structure, one might surmise that some of the items of the BSQ are redundant, thus the shorter versions—which retain a good reliability and discriminant capacity—can be used without loss of information.

Although developed to be applied to samples of patients with anorexia and bulimia nervosa, the BSQ-34 is flexible enough, in its detailing of the construct, to be used in samples of patients who may present body dissatisfaction in the absence of an eating disorder, such as patients with obesity, patients with body dysmorphic disorder, or patients looking for cosmetic or plastic surgery [74–79]. It remains advisable to use the longer, original version of the tool in the clinical setting since the detailed definition of the construct allows a more precise estimation of it in the candidate. However, in busy settings and for research purposes, some shorter version might be used.

The BSQ-34 was primarily developed for female patients; thus, some of its items might not be apt to measure body dissatisfaction in men (e.g., item 9 “Has being with thin women made you feel self-conscious about your shape?”; item 12, “Have you noticed the shape of other women and felt that your shape compared unfavorably?”; item 25, “Have you felt that it is not fair that other women are thinner than you?”). This may influence the comparability of scores in samples including both women and men, and, indeed, measurement invariance failed to be confirmed in some studies [19, 80, 81]. However, some adjustments might be done changing the wording of the items (substituting “women” for “men”). Moreover, some of the shorter versions of the BSQ-34 (8C and 8D) are gender non-specific; thus, they may be used with both men and women. Nevertheless, the wording is not the only factor involved in the measurement invariance failure by gender. Indeed, there is some evidence that the perception of thighs, stomach, and hips shows differential functioning across genders [82]. Moreover, men show a peculiar dissatisfaction with their muscularity and height [83], and with the aim of measuring this specific aspect of body dissatisfaction in men, some tools have been devised, such as the McCreary and Sasse’ Drive for Muscularity Scale [84], the Yelland and Tiggemann’s Drive for Muscularity Scale [85], or the Male Body Dissatisfaction Scale [86], also available in the Italian language [87]. Overall, caution is advised in comparing the scores of the BSQ-34 or its shorter versions between men and women. Measurement invariance of gender-adapted versions of the BSQ is a topic for further investigation in future studies.

### Strengths and limitations of the study

State-of-the-art statistics were used to analyze the study, and this is its major strength. However, several limitations should be also taken into account. The study included a large preponderance of female participants; thus, we cannot be certain that the excellent psychometric properties of the tool are generalizable to male samples. Most patients had anorexia nervosa; thus, we were unable to detail the distribution of the scores of the BSQ-34 in patients with bulimia nervosa or binge eating disorder. We had a small sample of controls, large enough to test the criterion validity but too little to apply the measurement invariance via CFA. Thus, we cannot state with certainty that the single-factor structure of the BSQ is invariant between cases and controls. Moreover, the controls were not perfectly matched to the patients, they were on average younger and better educated.

### What is already known on this subject?

The original BSQ-34 possesses good psychometric properties [18, 26, 30], which were confirmed for several of its translations [20, 27, 28]. Some of these psychometric properties, such as good reliability in the tested samples, were retained by its shorter versions [30, 88]. However, no formal evaluation of the psychometric properties of the Italian BSQ-34 has been done so far.

### What does this study add?

This study confirms the good psychometric properties of the Italian BSQ-34 and of its shorter versions, and proves its criterion validity, with a high positive diagnostic likelihood ratio (> 5) and a better capacity of identifying people with an eating disorder rather than excluding its presence.

## Conclusion

The Italian version of the BSQ possesses good psychometric properties—in both long and shortened versions—and it can be applied to measure body dissatisfaction for both clinical and research purposes. Further investigation of the discriminant properties of the tool with a larger sample of controls might be helpful.

## Supplementary Information

Below is the link to the electronic supplementary material.

Supplementary file1 (DOCX 22 KB)

## Data Availability

The corresponding author had full access to all the data in the study and takes responsibility for the integrity of the data and the accuracy of data analysis. Data sharing is not applicable since consent to publish was for aggregated data only.

## Code availability

Standard codes were used in the analysis. Sample codes will be shared upon demand.
