# Supplementary material for: Psychometric properties of the Italian body shape questionnaire: an investigation of its reliability, factorial, concurrent, and criterion validity
Source: Eat Weight Disord. 2022 Nov 9;27(8):3637–48. doi: 10.1007/s40519-022-01503-6 (PMC9803762; doi:10.1007/s40519-022-01503-6)
Supplement: Supplementary file 1 — Supplementary file1 (DOCX 22 KB) [file 40519_2022_1503_MOESM1_ESM.docx]

Supplementary Material

Table A1. Descriptive statistics for the BSQ items for the patients sample (n = 231).

| Items | Mean (SD) | Factor  loading |
| --- | --- | --- |
| 1. Il sentirti annoiato/a ti fa rimuginare sul tuo aspetto? | 3.8 (1.8) | 0.724 |
| 1. Ti sei preoccupato/a del tuo aspetto fisico tanto da ritenere di doverti mettere a dieta? | 3.8 (1.9) | 0.858 |
| 1. Hai mai pensato che le tue cosce, fianchi o sedere siano troppo grandi rispetto al resto del tuo corpo? | 3.8 (2.1) | 0.823 |
| 1. Ti sei preoccupato/a di poter ingrassare (o diventare più grasso/a)? | 4.6 (1.8) | 0.812 |
| 1. Ti sei preoccupato/a perché il tuo corpo non è abbastanza sodo? | 4.1 (1.8) | 0.680 |
| 1. Il sentirti pieno/a (es. dopo un pasto abbondante) ti fa sentire più grasso/a? | 4.5 (1.8) | 0.829 |
| 1. Il tuo aspetto fisico ti è sembrato così sgradevole da piangere? | 3.7 (1.9) | 0.726 |
| 1. Hai evitato di correre perché la tua carne potrebbe ballonzolare? | 1.9 (1.4) | 0.535 |
| 1. Lo stare insieme a donne magre ti ha fatto prendere coscienza dell'aspetto del tuo corpo? | 3.1 (1.8) | 0.429 |
| 1. Ti sei preoccupato/a perché, quando sei seduto/a, le tue cosce si distendono? | 3.5 (2.0) | 0.847 |
| 1. Mangiare anche una piccola quantità di cibo ti fa sentire grasso/a? | 3.5 (2.0) | 0.798 |
| 1. Hai osservato l'aspetto di altre donne e hai sentito che, in confronto a loro, il tuo aspetto è peggiore? | 4.1 (1.7) | 0.588 |
| 1. Lo stare a pensare al tuo aspetto ha interferito con la tua capacità di concentrazione (mentre guardi la TV, leggi, ascolti una conversazione)? | 3.9 (1.8) | 0.753 |
| 1. Essere nudo/a, come quando fai il bagno, ti fa sentire grasso/a? | 3.4 (2.0) | 0.859 |
| 1. Hai evitato di indossare vestiti che mettono in risalto l'aspetto del tuo corpo? | 3.9 (1.7) | 0.560 |
| 1. Hai immaginato di tagliar via le parti più grasse del tuo corpo? | 3.3 (1.9) | 0.768 |
| 1. Mangiare dolciumi, pasticcini o altri cibi ricchi di calorie ti fa sentire grasso/a? | 4.6 (1.8) | 0.787 |
| 1. Hai rinunciato a occasioni sociali (es. un party) perché il tuo aspetto fisico ti sembrava sgradevole? | 3.3 (1.9) | 0.651 |
| 1. Ti sei sentito troppo grosso/a o ingrassato/a? | 3.9 (1.9) | 0.880 |
| 1. Hai provato vergogna del tuo corpo? | 4.3 (1.7) | 0.730 |
| 1. Stai a dieta perché sei scontento/a del tuo corpo? | 3.7 (2.0) | 0.801 |
| 1. Ti sei sentito/a più soddisfatto/a del tuo aspetto quando il tuo stomaco era vuoto? | 4.1 (1.9) | 0.782 |
| 1. Hai pensato che l'aspetto che ti ritrovi è dovuto alla tua mancanza di autocontrollo? | 3.8 (1.8) | 0.511 |
| 1. Ti dà fastidio che gli altri vedano i rotoli di carne sul tuo addome o sul tuo stomaco? | 3.9 (2.1) | 0.857 |
| 1. Hai pensato che non è giusto che altre donne siano più magre di te? | 3.4 (2.0) | 0.702 |
| 1. Hai vomitato per sentirti più magro/a? | 2.4 (2.0) | 0.475 |
| 1. Quando sei in compagnia, ti sei preoccupato/a perché occupavi troppo spazio (es. sedendo su un divano o su un sedile dell'autobus)? | 2.5 (1.9) | 0.688 |
| 1. Ti dà fastidio che il tuo grasso formi delle fossette? | 3.3 (2.0) | 0.761 |
| 1. Guardare la tua immagine (es.in uno specchio o nelle vetrine di un negozio) ti fa sembrare sgradevole il tuo aspetto fisico? | 4.0 (1.8) | 0.728 |
| 1. Hai pizzicato delle zone del tuo corpo per vedere quanto erano grasse? | 3.7 (1.9) | 0.800 |
| 1. Hai evitato situazioni nelle quali gli altri potevano vedere il tuo corpo (spogliatoi pubblici, piscine)? | 3.7 (1.8) | 0.583 |
| 1. Hai assunto lassativi per sentirti più magro/a? | 2.2 (1.8) | 0.411 |
| 1. Sei stato/a particolarmente cosciente del tuo aspetto fisico quando eri in compagnia delle altre persone? | 3.5 (1.6) | 0.248 |
| 1. La preoccupazione per il tuo aspetto ti ha fatto pensare che dovresti fare attività fisica? | 4.2 (1.9) | 0.768 |
